# Impaired Motor Coordination and Learning in Mice Lacking Anoctamin 2 Calcium-Gated Chloride Channels

**DOI:** 10.1007/s12311-017-0867-4

**Published:** 2017-05-23

**Authors:** Franziska Neureither, Katharina Ziegler, Claudia Pitzer, Stephan Frings, Frank Möhrlen

**Affiliations:** 1 Department of Animal Molecular Physiology, Centre for Organismal Studies, Heidelberg University, Im Neuenheimer Feld 504, 69120 Heidelberg, Germany; 2 Interdisciplinary Neurobehavioral Core (INBC), Heidelberg University, Im Neuenheimer Feld 515, 69120 Heidelberg, Germany

**Keywords:** Purkinje cells, Inhibition, Plasticity, Calcium, Motor performance

## Abstract

Neurons communicate through excitatory and inhibitory synapses. Both lines of communication are adjustable and allow the fine tuning of signal exchange required for learning processes in neural networks. Several distinct modes of plasticity modulate glutamatergic and GABAergic synapses in Purkinje cells of the cerebellar cortex to promote motor control and learning. In the present paper, we present evidence for a role of short-term ionic plasticity in the cerebellar circuit activity. This type of plasticity results from altered chloride driving forces at the synapses that molecular layer interneurons form on Purkinje cell dendrites. Previous studies have provided evidence for transiently diminished chloride gradients at these GABAergic synapses following climbing fiber activity. Electrical stimulation of climbing fibers in acute slices caused a decline of inhibitory postsynaptic currents recorded from Purkinje cells. Dendritic calcium-gated chloride channels of the type anoctamin 2 (ANO2) were proposed to mediate this short-term modulation of inhibition, but the significance of this process for motor control has not been established yet. Here, we report results of behavioral studies obtained from *Ano2* ^*−/−*^ mice, a mouse line that was previously shown to lack this particular mode of ionic plasticity. The animals display motor coordination deficits that constitute a condition of mild ataxia. Moreover, motor learning is severely impaired in *Ano2* ^*−/−*^ mice, suggesting cerebellar dysfunction. This reduced motor performance of *Ano2* ^*−/*−^ mice highlights the significance of inhibitory control for cerebellar function and introduces calcium-dependent short-term ionic plasticity as an efficient control mechanism for neural inhibition.

## Introduction

The cerebellum controls body movements and mediates the acquisition of procedural memory [1]. The output neurons of the cerebellar cortex, the Purkinje cells, receive inhibitory GABAergic synapses from a network of molecular layer interneurons (MLIs), which provide fast feed-forward inhibition and co-determine the activity of the Purkinje cells [2–6]. MLIs limit the time window for synaptic integration in Purkinje cells to 1–2 ms [2], a control function that shapes the output signal of the cerebellar cortex. The MLI-Purkinje cell synapses are also subject to modulatory mechanisms, which probably contribute to motor learning. In particular, Purkinje cell depolarization that results from the activation of climbing fibers causes a slow but persistent increase of GABAergic synaptic strength termed rebound potentiation [7–9]. In genetic experiments, ablation of the γ2 subunit of GABA_A_ receptors at the MLI-Purkinje cell synapse impaired inter-limb coordination and motor memory consolidation [10, 11]. Moreover, pharmacologically boosted inhibition at this synapse severely disturbed motor coordination [12]. Thus, the inhibitory regulation of Purkinje cells by MLIs is clearly crucial for motor performance. Recent studies have indicated that the inhibition efficacy at the MLI-Purkinje cell synapse can be attenuated by a postsynaptic process which is triggered by activation of climbing fibers [13, 14]. Ca^2+^ signals that accompany climbing fiber-induced depolarization cause the opening of chloride channels of the type anoctamin 2 (ANO2, alias TMEM16B) [15]. It was proposed that these channels, in combination with increased Cl^−^ uptake through the co-transporter NKCC1, cause the local intra-dendritic Cl^−^ concentration to increase. Such postsynaptic Cl^−^ accumulation would reduce the inward driving force for chloride ions at MLI-Purkinje cell synapses and, hence, induce a decline of inhibitory postsynaptic current (IPSC) amplitudes. This mechanism was termed *depolarization-induced depression of inhibition* (DDI) and was shown to attenuate the inhibitory input for several minutes after climbing fiber activation [13]. This mode of modulation was detected by recording from Purkinje cells in tissue slices of rats [13] and mice [14]. Climbing fiber activity triggered a rapid, transient decline of IPSCs at the MLI-Purkinje cell synapse, and no such effect was observed in *Ano2* ^*−/−*^mice. This finding left the question unanswered whether the lack of DDI would compromise motor coordination and learning in *Ano2* ^*−/−*^mice. Thus, in the present study, we looked for the relevance of DDI for motor control. We compared the motor performance of wild-type and *Ano2* ^*−/−*^ mice in a variety of behavioral tasks designed to specifically reveal cerebellar dysfunction. We found that *Ano2* ^*−/−*^ mice display deficiency in motor coordination and motor learning. Our results illustrate the behavioral significance of calcium-dependent modulation of inhibitory network activity through short-term ionic plasticity, a novel pathway for controlling network function in the brain.

## Materials and Methods

### Animals: Housing, General Health, and Behavior

C57BL/6N mice (Charles River Laboratories, Germany) and *Ano2* ^*−/−*^ mice [16] (kindly provided by Thomas Jentsch, Leipniz-Institute for Molecular Pharmacology, Berlin) were kept in groups of 2–3 with ad libitum access to food and water. A standard 12-h light/dark cycle was provided (light on: 7 am to 7 pm) and temperature was maintained at ±22 °C at a relative humidity of 40–50%. All experiments were approved by the Regierungspräsidium Karlsruhe and were in agreement with national and international guidelines. For general health screening, a modified version of the SHIRPA test [17] was used for both genotypes. Spontaneous activity, anxiety, body strength, and muscle tone as well as several reflexes were tested according to the provided scoring system. General behavior was monitored using the *LABORAS* animal behavior observation system (Metris B.V., Netherlands) individually for six mice of each genotype for 72 h. Recorded activities included locomotion, climbing, rearing, grooming, eating, and drinking.

### Experimental Design

Following a habitation time of 1 week, animals were handled for three consecutive days before the behavioral tests were started. Behavioral tests were performed by one female experimenter only in order to avoid unnecessary stress for the mice. Tests were run, if not indicated otherwise, from 1 to 4 pm. Non-automatic rated experiments (apart from the SHIRPA test) were analyzed in a blind fashion by two different persons. Only male, adult mice were used with a starting age of 8 weeks (cohort 3) and 10–12 weeks (cohorts 1 and 2) and tests were conducted for up to 10 weeks (cohorts 1 and 2) or 4 days (cohort 3). Numbers of animals tested were (wt/*Ano2* ^*−/−*^) general behavior 6/6 (cohort 1); rotarod 9/8 (cohort 1); open field, grip strength, parallel rod floor, gait analysis 9/10 (cohort 2); horizontal ladder 9/9 (cohort 2); and voluntary wheel running 4/5 (cohort 3).

### Immunohistochemistry

To prepare cerebellar cryosections, animals were anesthetized by isoflurane (Baxter, Germany) inhalation and killed by an overdose of isoflurane. The cerebellum was removed and the fresh, unfixed tissue was cut on ice in phosphate-buffered saline (PBS; 130 mM NaCl, 8.1 mM Na_2_HPO_4_, 1.9 mM NaH_2_PO_4_, pH 7.4).) using a vibratome (VT1000S, Leica Biosystems, Wetzlar, Germany). Sections of 180 µm thickness were fixed with 2% paraformaldehyde (PFA) for 30 min and washed 4× 10 min with PBS (for ANO2 immunostaining) or with PBST (PBS containing 0.5% Tween 20 (Carl Roth, Germany, 9127.1)) for calbindin immunostaining. For immunofluorescence staining, sections were incubated in blocking solution (goat serum, Sigma-Aldrich, Germany, G9023) for 2 h and then the primary antiserum (diluted in blocking solution) was applied overnight at room temperature. After washing 4× 10 min with PBS or PBST, the secondary antisera, conjugated with fluorescence tags, were incubated for 2 h and washed 4× 10 min with PBST. To visualize cell nuclei, the slices were incubated in 0.3 μM DAPI (Sigma-Aldrich, 32670) for 15 min, washed 4× 5 min in PBS, and then mounted on glass slides with Aqua-Poly/Mount (Polyscience, Germany, 18606). The sections were kept on a shaker for the whole staining process where they were gently shaken to improve the uptake of the antisera. The primary antibody against ANO2 (used at 1:200) was raised in guinea pig in our lab and was extensively characterized in various tissues including cerebellum [14, 18–20]. The primary antibody against calbindin (dilution 1:500) was raised in rabbit (Swant, Marly, Switzerland, CB-38a) and binds specifically to calbindin D-28k. The secondary antibodies were donkey anti-rabbit conjugated with Alexa Fluor 568 (Invitrogen, A10042, dilution 1:1000) and goat anti-guinea pig conjugated with Alexa Fluor 488 (Invitrogen, A11073).

### Grip Strength Analysis

Forelimb strength of both mouse lines was tested using a grip strength meter (Ugo Basile, Gemonio, Italy, model 47106), which automatically measured the force needed for the mouse to release its grip. A mouse had to grasp a trapeze wire and would cling to it until the pulling force of the experimenter exceeded its own pulling strength. After an initial training session, the mice were tested in one session only, in which each mouse was tested in three consecutive runs. The runs were then averaged for each animal.

### Open Field Test

Mice were individually placed in a wooden box (40 × 40 cm) for a period of 5 min and their movements were camera tracked. Only one run per mouse was conducted and the distance they walked was analyzed to evaluate their spontaneous activity.

### Parallel Rod Floor Test

The open field setup was adjusted with an additional rod floor with parallel bars (3 mm in diameter, 6 mm space in between) to attain the mice’s locomotor activity at slightly aggravated conditions. On three consecutive days, mice were placed on the rod and their movement was video-tracked over a period of 5 min. One session per day was conducted per mouse. The maximal distance walked by all mice of a genotype was pooled for each session.

### Horizontal Ladder Test

A horizontal ladder (40 cm in length, 43 rung positions) with a randomized and irregular rung pattern was used to assess skilled motor coordination of the animals. The metal rungs were 3 mm in diameter with a minimal space of 6 mm between two rods. Rods were placed in an irregular randomized pattern and their pattern was not altered during the testing phase. An initial training session was conducted to let the mice get acquired to the setup. While testing, mice were put at the beginning of the ladder individually and had to cross the ladder voluntarily. As a reward input, their home cage with the remaining housing companions was placed at the end of the ladder. Every walk was video-taped and the video was stopped as soon as the mice reached the end of the ladder. Each mouse did four trails so that each body side was taped twice. The videos were then analyzed by two different evaluators in a blinded manner. The time the mice needed to traverse the ladder was measured and the number of steps counted. Final results were obtained by averaging four runs of each mouse as well as the individual results of the two evaluators.

### Gait Analysis

The animals’ gait patterns were analyzed using the CatWalk XT analysis apparatus (Noldus, Netherlands, version 10.6). The 1.3 m black corridor led over a glass plate that was illuminated by a green LED light. As soon as the mouse’s paw touched the glass slide, this green light was reflected and the resulting illuminated green footprint could then be captured by a high speed camera underneath the glass. As the mouse traversed the glass plate, its gait pattern was recorded and the resulting data automatically were analyzed by the CatWalk XT software according pre-set paradigms [21]. Evaluation parameters used for our analysis are specified in the legend to Table 1.

**Table 1:** Results of gait analysis with significant differences between wild-type (*n* = 9) and *Ano2* ^*−/−*^ mice (*n* = 10)

Parameter	*Ano2* ^+/+^	*Ano2* ^*−/−*^	*p* value
Basic Locomotion			
Average running speed (cm/s)	17.60 ± 1.14	11.84 ± 0.64	<0.001
Number of steps per s	12.35 ± 0.57	9.70 ± 0.39	<0.001
Total number of steps	84.23 ± 3.06	95.75 ± 2.19	<0.01
Running duration (s)	3.48 ± 0.26	4.89 ± 0.25	<0.001
Step characteristics			
Stride length (cm)^a^	5.28 ± 0.10	4.68 ± 0.07	<0.001
Step cycle for each paw (s)^b^	0.32 ± 0.009	0.42 ± 0.01	<0.001
% of step cycle standing	56.86 ± 0.53	61.18 ± 0.57	<0.001
Swing speed in cycle (cm/s)	45.58 ± 1.09	37.34 ± 0.75	<0.001
Mean duration of standing (s)^c^	0.20 ± 0.009	0.28 ± 0.007	<0.001
Hind paws: single stance (s)^d^	0.11 ± 0.003	0.13 ± 0.004	<0.01
Hind paws: dual stance (s)^e^	0.04 ± 0.004	0.07 ± 0.006	<0.001
Distance HP-FP: left (cm)^f^	1.10 ± 0.08	1.44 ± 0.12	<0.05
Distance HP-FP: right (cm)	0.99 ± 0.10	1.44 ± 0.07	<0.001
Width between hind paws (cm)	2.45 ± 0.04	2.59 ± 0.04	<0.05
Support on three legs (%)^g^	22.60 ± 1.94	31.12 ± 1.11	<0.001
Support on diagonal legs (%)^h^	60.81 ± 3.17	46.65 ± 2.5	<0.001
Gait variability			
Run maximum variation (%)^i^	55.00 ± 4.86	68.65 ± 3.89	<0.05
Step sequence regularity (%)^j^	88.80 ± 1.15	83.53 ± 1.74	<0.05
Interpaw coordination^k^			
RF->LH	0.68 ± 0.03	0.54 ± 0.04	<0.05
LF->RH	0.73 ± 0.03	0.56 ± 0.03	<0.001
LH->RH	0.57 ± 0.03	0.40 ± 0.04	<0.01
LF->RF	0.80 ± 0.02	0.72 ± 0.02	<0.01
RF->RH	0.68 ± 0.03	0.53 ± 0.03	<0.001
LF->LH	0.68 ± 0.04	0.55 ± 0.03	<0.01

^a^Distance between successive placements of the same paw

^b^Time between two consecutive initial placements of the same paw

^c^Duration of ground contact of paw

^d^Duration of ground contact of single hind paw

^e^Duration of simultaneous ground contact of two hind paws

^f^Distance between hind paw and ipsilateral front paw in the same step cycle

^g^Percentage of time with three paws on the ground

^h^Percentage of time with contralateral hind paw—front paw combinations on the ground

^i^Variation in running speed given as percentage of average running speed

^j^A measure of uniformity in the execution of stepping patterns (100% indicates a completely uniform running pattern)

^k^Temporal relationship between placement of the indicated paw pairs; the range of the indicated Cstat parameter R (strength of directedness) is between 0 and 1, with smaller values indicating larger variance of phase dispersion

### Voluntary Wheel Running

To monitor voluntary wheel running activity, mice were placed individually in a home cage with a running wheel (diameter 9.2 cm, width 5.1 cm) to which they had free access. Water and food was provided, and the experiment was conducted at a 12 h light/dark cycle during four successive days and nights. Wheel revolutions per 15 min intervals were recorded by a sensor connected to a computer interface (Columbus Instruments, Columbus, OH, USA). Only mice that did run were taken into account.

### Rotarod

Accelerated rotarod tests (4–40 rpm) were performed up to maximal 8 min to examine the animals’ motor coordination and motor learning skills. The rotarod apparatus (Ugo Basile, Gemonio, Italy, model 47600) had a rod of 3 cm in diameter and five lanes with a width of 5 cm each. The test is designed to measure the time mice are able to run on the accelerated rod. As soon as a mouse falls off the rod, the time is stopped automatically. After an initial training session, mice were tested for three consecutive weeks every 2–3 days. In each session, mice were tested in three trials with a recovery phase of 10 min in between. For analysis, the three trials were averaged for each mouse. After a test break of 3 weeks, mice were again tested for 3 weeks in the same pattern.

## Results

**Fig. 1:**
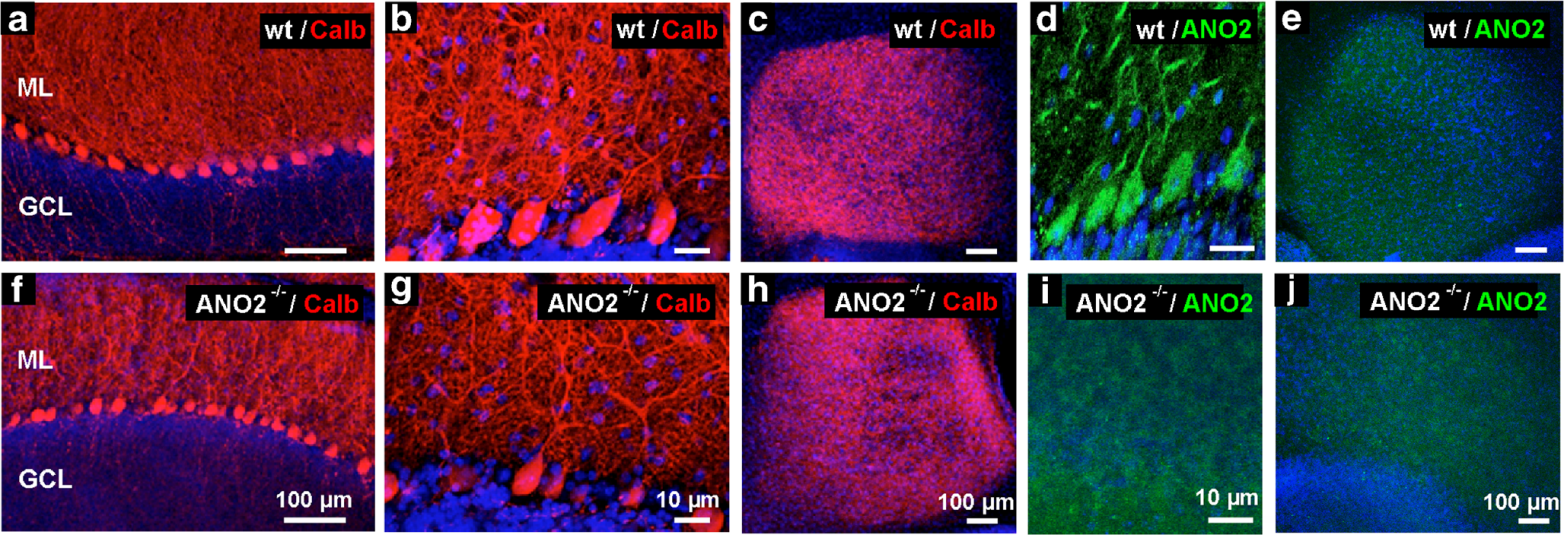
Anatomical integrity of cerebellar cortex and deep cerebellar nuclei in *Ano2* ^*−/−*^ mice. **a** Overview of the cerebellar cortex of a wild-type mouse with calbindin immunostaining (*red*) and DAPI nuclear stain (*blue*), depicting the molecular layer (*ML*), the granule cell layer (*GCL*), and, in between, a row of Purkinje cell somata. **b** Structural details of calbindin-expressing Purkinje cells with their dendrites in the molecular layer. Nuclei of molecular layer interneurons are visible by DAPI stain. **c** Calbindin-staining of a deep cerebellar nucleus in a wild-type mouse. **d** ANO2-immunosignals in Purkinje cell somata and dendrites. **e** Lack of specific immunostain in a deep cerebellar nucleus of a wild-type mouse. **f–j** Immunostains against calbindin (*red*) and ANO2 (*green*) as in the upper row of images, but with cryosections from *Ano2* ^*−/−*^ mice. *Calibration bars* are 10 μm in **b**, **g**, **d**, and **i** and 100 μm in all others

*Ano2* ^*−/−*^ mice that lacked DDI in acute tissue slices [14] were used for these experiments. The animals displayed normal cerebellar anatomy without conspicuous structural differences in the granule cell layer, the Purkinje cell layer, the molecular cell layer, and the deep cerebellar nuclei (wt: Fig. 1a–c; *Ano2* ^*−/−*^: Fig. 1f–h). In accordance with previous data [14], ANO2 expression was detected in Purkinje cells (Fig. 1d, i) but not in the deep cerebellar nuclei (Fig. 1e, j). During home cage observation, *Ano2* ^*−/−*^ mice showed normal basal voluntary activities, including similar locomotion, climbing, rearing, grooming, eating, and drinking. Mean body weight increased during 6 weeks of experimentation from 25.9 ± 0.3 to 30.4 ± 0.6 g in wild-type mice and from 28.3 ± 0.7 to 31.1 ± 0.4 g in *Ano2* ^*−/−*^animals. In open field tests, they displayed similar explorative behavior and covered similar distances during the 5-min test period (wt 2.04 ± 0.15 m; *Ano2* ^*−/−*^1.79 ± 0.18 m, *t* test, *p* = 0.31). Furthermore, the grip strength test yielded similar results for muscle strength in the front limbs (wt 523 ± 32 mN; *Ano2* ^*−/−*^ 574 ± 20 mN; *n* = 9, *t* test, *p* = 0.19), indicating normal muscle development. *Ano2* ^*−/−*^ mice scored normally in SHIRPA tests for spontaneous activity, anxiety, body strength, muscle tone, and various reflexes, with the exception of the negative geotaxis test where they displayed a reduced tendency to climb upward on a vertical grid. Thus, *Ano2* ^*−/−*^ mice appeared basically healthy and active.

**Fig. 2:**
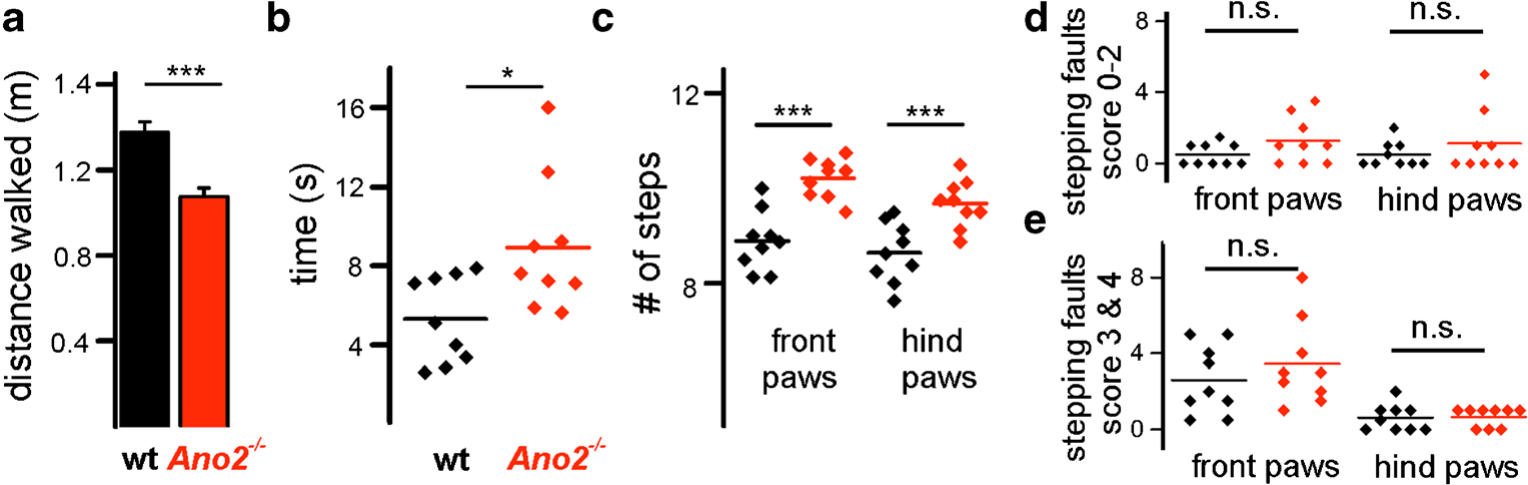
*Ano2* ^*−/−*^ mice display gait coordination problems when walking on grids. **a** *Ano2* ^*−/−*^ mice (*red*) covered shorter distances during voluntary walking on a parallel rod floor (wt 1.38 ± 0.05 m; *Ano2* ^*−/−*^ 1.08 ± 0.04 m; two-way ANOVA, *p* < 0.001, Bonferroni correction). **b** On the horizontal ladder with several missing rungs, *Ano2* ^*−/−*^ mice (*red*) needed more time to cross the 40-cm-long ladder (wt 5.3 ± 0.7 s; *Ano2* ^*−/−*^ 8.9 ± 1.1 s; Student’s *t* test, *p* = 0.016). **c** *Ano2* ^*−/−*^ mice (*red*) also needed more steps to walk over the horizontal ladder, with significant differences in both front paws (wt 8.9 ± 0.2 steps; *Ano2* ^*−/−*^ 10.2 ± 0.13 steps; Student’s *t* test, *p* < 0.001) and hind paws (wt 8.6 ± 0.2 steps; *Ano2* ^*−/−*^ 9.7 ± 0.16 steps; Student’s *t* test, *p* = 0.001). **d**, **e** Analysis of paw placement on the horizontal ladder revealed no significant differences between wild-type and *Ano2* ^*−/−*^ mice (*n* = 9; all *t* test *p* values > 0.05). Scoring corresponds to a standard foot fault scoring system [23]. Scores 0–2 represent various patterns of slipping from a rung, while scores 3–4 depict corrective movements following a faulty step. Mean values from nine wild-type and ten *Ano2* ^*−/−*^ mice are displayed ± SEM; *p < 0.05, ***p < 0.001

**Fig. 3:**
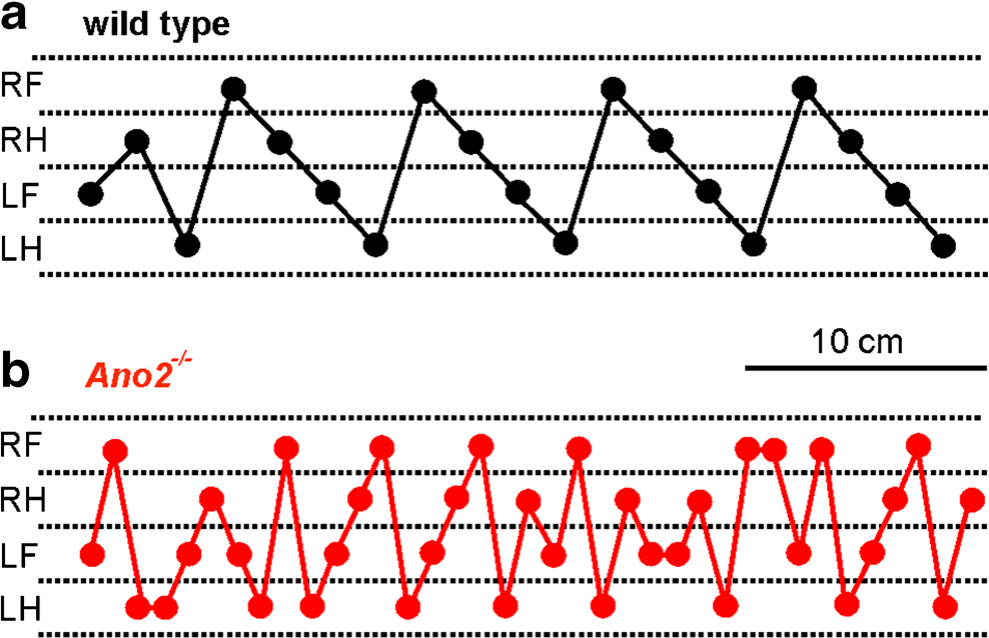
Walking patterns of *Ano2* ^*−/−*^ mice differ from wild types. **a** Regular walking pattern of a wild-type mouse as recorded by the Catwalk XT gait analysis system. The mouse walked the 36-cm test lane within 20 s. Paw positions are indicated as *dots* for the right forepaw (*RF*), right hindpaw (*RH*), left forepaw (*LF*), and left hindpaw (*LH*). **b** Representative walking pattern of an *Ano2* ^*−/−*^ mouse that walked along the same test lane in 27 s. The results of a detailed gait analysis are given in Table 1

To specifically examine motor coordination abilities, we first used the parallel rod floor test [22]. Mice walked spontaneously and voluntarily on a grid of steel bars while being video-taped. The distance that the animals covered in three 5-min sessions was significantly shorter in *Ano2* ^*−/−*^ mice (Fig. 2a). In the related horizontal ladder test, mice had to walk over a series of 27 rungs, with 16 randomly positioned gaps. *Ano2* ^*−/−*^ mice required significantly more time (Fig. 2b) and more steps (Fig. 2c) to perform this task. Although *Ano2* ^*−/−*^ mice appeared insecure and cautious on the horizontal ladder, their forelimb and hindlimb placement was not significantly impaired. The number of slips (Fig. 2d) and corrective paw movements (Fig. 2e) was similar to wild-type animals. To closer inspect walking parameters for differences between wild-type and *Ano2* ^*−/−*^ mice, we used the CatWalk XT gait analysis system [21]. In this voluntary walking assay, the position of each paw print was recorded and was analyzed with respect to walking patterns and leg coordination. Walking patterns of wild-type mice were typically regular and repetitive (Fig. 3a), while *Ano2* ^*−/−*^ mice displayed a more irregular gait when walking along the test lane (Fig. 3b). Table 1 summarizes those parameters of the quantitative gait analysis that differed significantly between wild-type and *Ano2* ^−/−^ mice. Briefly, the data set indicates that locomotion was slower in *Ano2* ^*−/−*^ mice (*basic locomotion* in Table 1), that stepping patterns and stepping speed were different (*step characteristics* in Table 1), that *Ano2* ^*−/−*^ mice displayed reduced gait regularity (*gait variability* in Table 1), and that the coordination of paw positioning was compromised (*interpaw coordination* in Table 1). Several of these differences point to cerebellar problems in *Ano2* ^*−/−*^ mice. For example, the increased width between hindpaws is indicative of ataxia that does not depend on walking speed [24]. The increased gait variability is consistent with an earlier report on a cerebellar small-lesion model [25]. Taken together, the gait analysis produced indications of mild ataxia during spontaneous, voluntary walking in *Ano2* ^*−/−*^ mice, a condition that appeared to enforce a slow, hesitant walking pattern.

**Fig. 4:**
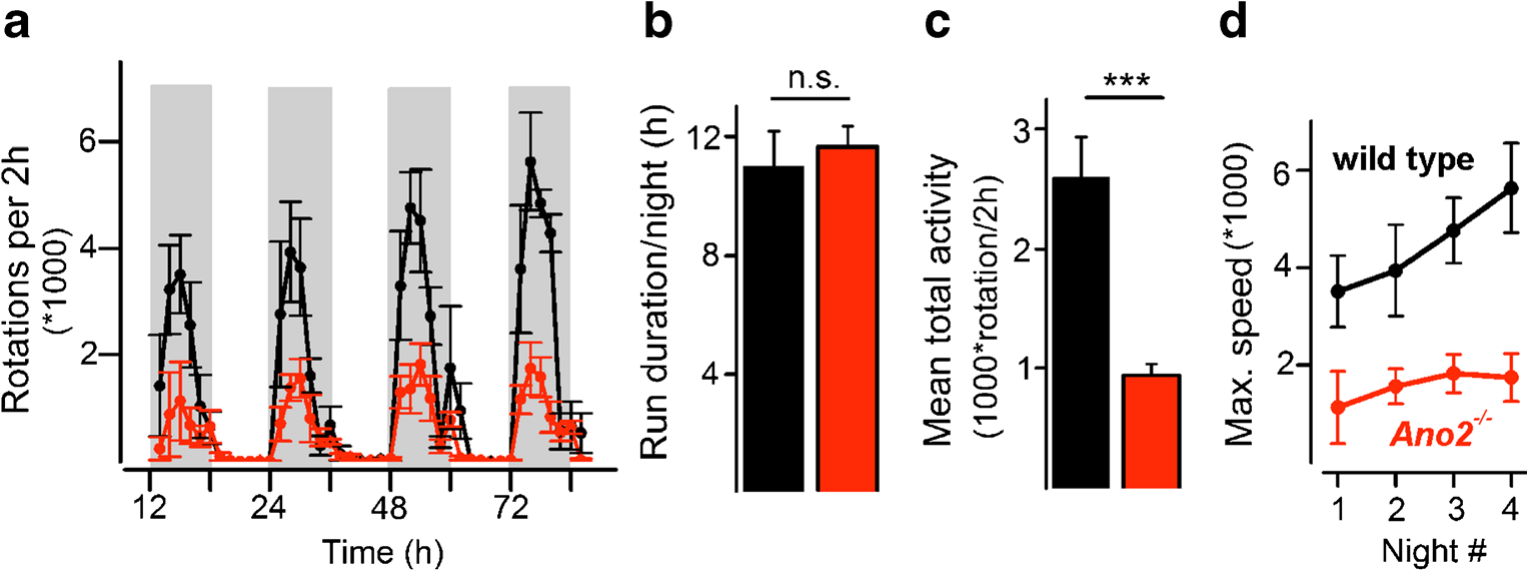
Impaired voluntary motor performance in *Ano2* ^*−/−*^ mice. **a** Both wild-type (*black*) and *Ano2* ^*−/−*^ mice (*red*) used running wheels for nocturnal activity. The *shaded boxes* depict the dark periods from 7 pm to 7 am during the 84-h experiment. Plotted are the rounds accumulated over successive 2-h intervals. **b** Both mouse lines used the wheel for similar durations within the active period, proving comparable motivation for the task. **c** Over the four nights of activity, *Ano2* ^*−/−*^ mice ran on average fewer rounds on the wheel than wild-type animals (wt 2599 ± 336 rotations/2 h; *Ano2* ^*−/−*^ 939 ± 95.6 rotations/2 h; two-way ANOVA, *p* < 0.001, Bonferroni correction). **d** The motor performance on the running wheel increased in wild-type but not in *Ano2* ^*−/−*^ mice. Two-way ANOVA: genotypes, *p* < 0.001; time dependence, *p* = 0.11; interaction, *p* = 0.2. Mean values from four wild-type and five *Ano2* ^*−/−*^ mice are displayed ± SEM; ***p < 0.001

To examine whether motor learning was also affected in the *Ano2* ^*−/−*^ mice, each mouse was provided with a running wheel over a test period of 4 days and nights. Both wild-type and *Ano2* ^*−/−*^ mice used the wheels during the night over a total period of 10–12 h with only short intermissions (Fig. 4a). Both mouse lines spent similar time on wheel running, displaying equally strong motivation for this activity (Fig. 4b). *Ano2* ^*−/−*^ mice, however, reached 70% less rotations within the testing period (Fig. 4c). Moreover, while wild-type mice almost doubled their motor performance over four nights, *Ano2* ^*−/−*^ mice were unable to increase the number of rotations (Fig. 4d). These results indicate impaired motor coordination and learning in *Ano2* ^*−/−*^ mice in a voluntary locomotion task.

**Fig. 5:**
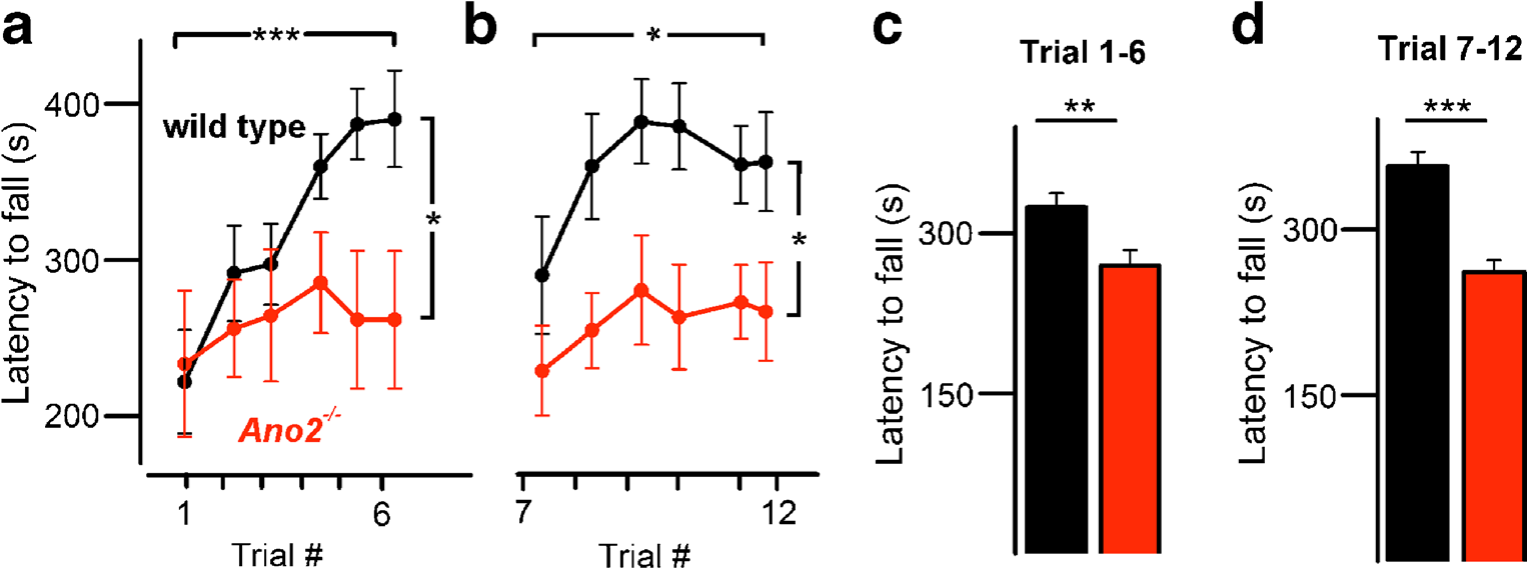
Impaired enforced motor performance and learning in *Ano2* ^*−/−*^ mice. **a** Motor performance on the accelerating rotarod increased in wild-type mice (*black*) over 6 days of testing (Student’s *t* test, *p* < 0.001), while *Ano2* ^*−/−*^ mice (*red*) did not improve significantly (*p* = 0.12). Two-way ANOVA: genotypes, *p* < 0.01; time dependence, *p* < 0.01; interaction, *p* = 0.38. **b** Wild-type mice rapidly regained motor performance levels on the rotarod after a 20-day intermission, indicating consolidated procedural memory. *Ano2* ^*−/−*^ mice displayed no significant motor learning ability. Two-way ANOVA: genotypes, *p* < 0.001; time dependence, *p* = 0.15; interaction, *p* = 0.93. **c** Statistical analysis of the latency to fall from rotarod (data from trials 1–6) illustrates a significantly reduced motor performance in *Ano2* ^*−/−*^ mice (wt 321.6 ± 13.3 s; *Ano2* ^*−/−*^ 270 ± 15 s; two-way ANOVA, *p* < 0.01, Bonferroni correction). **d** In the repeated rotarod session (data from trials 7–12), a persistent difference in motor performance was seen (wt 360 ± 12 s; *Ano2* ^*−/−*^ 261 ± 11 s; two-way ANOVA, *p <* 0.001, Bonferroni correction). Mean values from nine wild-type and eight *Ano2* ^*−/−*^ mice are displayed ± SEM; *p < 0.05, **p < 0.01, ***p < 0.001

To find out whether the animals were able to learn in an enforced locomotion task, we challenged them with the accelerating rotarod test. Both wild-type and *Ano2* ^*−/−*^ mice performed similarly well on the first day of testing (Fig. 5a). *Ano2* ^*−/−*^ mice, however, did not significantly improve their motor skills over 3 weeks of rotarod training, while wild-type mice showed a steady learning curve and almost doubled their time on the rotarod (Fig. 5a). To test whether this learned motor skill represented consolidated procedural memory, we repeated the entire experiment after a 20-day interval. Wild-type mice regained their full rotarod performance already on the second day of rotarod training, while *Ano2* ^*−/−*^ mice again did not improve significantly (Fig. 5b). These results demonstrate that *Ano2* ^*−/−*^ mice have a reduced ability for motor learning in both the initial (Fig. 5c) and the repeated (Fig. 5d) period of the experiment, corroborating the evidence for cerebellar dysfunction in this mouse line.

## Discussion

Cl^−^ concentrations in dendrites are not uniform but can change locally and transiently [26–33]. The results presented here are consistent with the idea that ANO2 channels modulate Purkinje cell inhibition by MLIs and that this modulation plays a role in motor coordination and motor learning. The behavioral protocols described here are suitable for the detection of cerebellar dysfunctions. Several mouse models, mostly animals with defect Purkinje cells, display a motor phenotype in these tasks [11, 34, 35]. We thus interpret the motor problems of *Ano2* ^*−/−*^ mice as indicative of a cerebellar problem. This view is supported by previous results from mice with genetically disabled MLI-Purkinje cell synapses. L7-Δγ2 mice do not express functional synaptic GABA_A_ receptors in Purkinje cells [10]. These animals tend to use smaller steps on the Erasmus Ladder, a sophisticated version of the horizontal-grid task [11], and generally display only subtle changes in gait, even less conspicuous than the walking pattern observed in *Ano2* ^*−/−*^ mice. In comparing these two mouse lines, one has to take into account that the effective removal of fast-forward inhibition in L7-Δγ2 mice constitutes a severe interference with cerebellar circuitry, which probably triggers developmental compensations to extenuate any phenotype [12]. In contrast, the removal of the modulatory pathway in *Ano2* ^*−/−*^ mice appears to be less severe, and its effects on motor performance are not obliterated by developmental compensation.

Taken together, the lack of DDI reported previously [14] and the *Ano2* ^*−/−*^ phenotype described here match the notion of dysregulation on the level of MLIs. ANO2 is not expressed in skeletal muscle, motoneurons, or other spinal cord neurons. Muscular dysfunction can, therefore, be ruled out as a cause for the *Ano2* ^*−/−*^ phenotype, a conclusion supported by the normal grip strength of *Ano2* ^*−/−*^ mice. Apart from the cerebellum, ANO2 channels are also expressed in the cilia of olfactory sensory neurons [16, 36, 37], in rod photoreceptor terminals [19, 38], in dendrites of hippocampal pyramidal neurons [39], and in thalamocortical neurons [40]. Contribution to the *Ano2* ^*−/−*^ phenotype from these structures still has to be examined. In olfaction, ANO2 ablation leads to a sensory phenotype that shows no altered sensitivity in operant conditioning experiments [16] but displays problems with the detection of novel odors [41]. Mice in our experiments were habituated to their olfactory surroundings and should, consequently, not be compromised in olfactory performance. No *Ano2* ^*−/−*^ phenotype was reported so far for the visual system. ANO2 channels seem to be involved in the regulation of glutamate release from rod terminals under scotopic conditions [19], but the contribution of ANO2 to visual performance is not yet understood. Whether the loss of ANO2 in hippocampal neurons may contribute to the motor problems of the *Ano2* ^*−/−*^ mice cannot be assessed at this stage. Hippocampal lesions tend to cause hyperactivity and do not reduce rotarod performance [42], whereas the *Ano2* ^*−/−*^ mice present the opposite phenotype. Thalamocortical neurons transmit sensory information from the thalamus to the cortex. They show a distinct, ANO2-mediated type of self-inhibition that reduces their spike frequency during sensory stimulation [40]. *Ano2* ^*−/−*^ mice respond more strongly to visceral—but not to acute—pain than wild-type mice, and it is conceivable that increased sensory perception may also affect motor performance. However, a possible link between thalamocortical self-inhibition and cerebellar function still needs to be investigated. A possible contribution to the *Ano2* ^*−/−*^ phenotype may come from the striatum, as striatal low-threshold spiking, NPY-positive interneurons express ANO2 and appear to require the channels for maintaining membrane potential oscillations of 3–7 Hz [43]. The inhibitory interneuron circuits of the striatum serve to filter incoming signals, and their dysfunction may cause problems in motor coordination [44]. A striatal aspect of the *Ano2* ^*−/−*^ phenotype must be examined in future when conditional ANO2 knockouts become available [45]. For the present, the global *Ano2* ^*−/−*^ mouse provides a consistent set of results linking mild ataxia and reduced motor learning to the lack of DDI in cerebellar Purkinje cells.

The attenuation of MLI input through DDI limits GABAergic inhibition of Purkinje cells after climbing fiber stimulation, an effect that may last for several minutes [13]. Our results indicate that the loss of this process in *Ano2* ^*−/−*^ mice causes problems in motor coordination as brought out by mildly ataxic behavior on horizontal grids, running wheels, and rotarods. However, motor memory consolidation also appeared compromised, as *Ano2* ^*−/−*^ mice seemed to be unable to improve motor performance significantly and could not maintain the small progress they achieved in rotarod training over 2 weeks. Such impaired procedural memory may be related to the ablation of ANO2 inasmuch as the reduced disinhibition of Purkinje cells in *Ano2* ^*−/−*^ mice attenuates the output signal that the cerebellar cortex conveys to the deep cerebellar nuclei. While this concept has not yet been tested experimentally, it is consistent with the notion that signal flow from Purkinje cells to cerebellar nuclei is necessary for memory consolidation [46]. As ANO2 appears not to be expressed in neurons of the cerebellar nuclei, the reduced motor learning ability of *Ano2* ^*−/−*^ mice is more likely caused by impaired communication between cerebellar cortex and nuclei than by functional problems in the nuclei themselves. In any case, ANO2 channels contribute to motor coordination and motor learning in the cerebellum.

## Conclusion

Behavioral experiments revealed mild ataxia and reduced motor learning in *Ano2* ^*−/−*^ mice, which lack the calcium-dependent mode of ionic plasticity termed *depolarization-induced depression of inhibition* at the MLI-Purkinje cell synapses. This finding demonstrates that modulation of inhibitory input to Purkinje cells is an important component of signal processing in the cerebellar cortex. Modulation appears to protect the cells from excessive inhibition during and shortly after an excitatory signal is delivered by a climbing fiber. It precedes the long-lasting (>1 h) increase of inhibitory strength termed *rebound potentiation*. Thus, a biphasic modulation of synaptic strength appears to shape the GABAergic inhibition of cerebellar Purkinje cells.
